# CD69^+^ memory T lymphocytes of the bone marrow and spleen express the signature transcripts of tissue‐resident memory T lymphocytes

**DOI:** 10.1002/eji.201847982

**Published:** 2019-01-30

**Authors:** Francesco Siracusa, Pawel Durek, Mairi A. McGrath, Özen Sercan‐Alp, Anna Rao, Weijie Du, Carla Cendón, Hyun‐Dong Chang, Gitta Anne Heinz, Mir‐Farzin Mashreghi, Andreas Radbruch, Jun Dong

**Affiliations:** ^1^ Cell Biology Deutsches Rheuma‐Forschungszentrum Berlin (DRFZ), a Leibniz Institute Berlin Germany; ^2^ Schwiete‐Laboratory for Microbiota and Inflammation Deutsches Rheuma‐Forschungszentrum Berlin (DRFZ), a Leibniz Institute Berlin Germany; ^3^ Therapeutic Gene Regulation Deutsches Rheuma‐Forschungszentrum Berlin (DRFZ), a Leibniz Institute Berlin Germany; ^4^ Experimental Rheumatology Charité University Medicine Berlin Berlin Germany

**Keywords:** Bone marrow, CD69, Memory T cells, Spleen, Tissue‐resident signature genes

## Abstract

It is a matter of current debate whether the bone marrow is a hub for circulating memory T lymphocytes and/or the home of resident memory T lymphocytes. Here we demonstrate for CD69^+^ murine CD8^+^, and CD69^+^ murine and human CD4^+^ memory T lymphocytes of the bone marrow, making up between 30 and 60% of bone marrow memory T lymphocytes, that they express the gene expression signature of tissue‐resident memory T lymphocytes. This suggests that a substantial proportion of bone marrow memory T lymphocytes are resident. It adds to previous evidence that bone marrow memory T cells are resting in terms of mobility and proliferation, and maintain exclusive long‐term memory to distinct, systemic antigens.

It is a matter of current debate, whether the bone marrow is a hub for circulating memory T lymphocytes, and/or the home of tissue‐resident memory T (T_RM_) lymphocytes. While several groups could not find evidence for tissue‐residency of bone marrow memory T cells 1, we have previously demonstrated the exclusive maintenance of T‐cell memory for distinct (systemic) antigens in the human and murine bone marrow, arguing that at least memory T cells maintaining those specificities were bone marrow‐resident 2, 3, 4. We have also shown that bone marrow memory T cells are resting in terms of proliferation 2, 3, 5 and are maintained independent of the circulation, i.e. their numbers are unaffected by treatment with FTY720 6, a drug that blocks egress of lymphocytes from secondary lymphoid organs by antagonizing of S1PR1. Moreover, in mice and humans approximately 30% to 60% of memory T cells of the bone marrow express the residency marker CD69, and for human bone marrow memory CD4^+^ T lymphocytes and murine bone marrow memory CD4^+^ and CD8^+^ T lymphocytes, we have shown that these cells do not express S1pr1 5, 7, reported to enable lymphocyte egress into the blood.

Here, we show that CD69 expressing murine memory CD4^+^ and CD8^+^ T cells, and human memory CD4^+^ T cells of the bone marrow also express a set of genes reported to be signature genes of T_RM_ from other tissues 8, 9. We isolated human memory CD4^+^ T cells and murine CD8^+^ memory T cells from bone marrow in steady‐state situations and murine CD69^+^ and CD69^−^ memory CD4 T cells from spleen and bone marrow 60 days after a secondary immunization with a defined antigen (LCMV GP_61–80_) 7. The isolation of the murine CD4^+^ memory T cells was carried out according to published guidelines 10 and is documented in Supporting Information Fig. 1. The isolation of murine memory CD8 and human memory CD4 has been documented earlier 3, 5. Transcriptomes of murine CD8^+^ and human CD4^+^ memory T cells were analyzed by Affymetrix microarrays, while transcriptomes of murine CD4 memory T cells were analyzed by next generation RNA‐Seq. Both CD4 (Fig. 1A) and CD8 (Fig. 1B) murine CD69^+^, but not CD69^−^ memory T cells of the bone marrow, show the “universal” transcriptional signature of T_RM_ cells reported by Mackay and colleagues for CD8 T_RM_ cells of different tissues (not bone marrow) 9. It should be noted that the Affymetrix microarrays lacked probes for the genes Zfp683 (Hobit), Sidt1, and A430078G23Rik (Fig. 1B). Interestingly, the murine T_RM_ signature genes were also expressed by the CD69^+^ CD4 memory T cells from the spleen (Fig. 1A). Human CD69^+^ but not CD69^−^ bone marrow memory CD4^+^ T cells show the T_RM_ signature previously reported for human CD4^+^ and CD8^+^ T_RM_ by Kumar and colleagues 8 (Fig. 1C). These differences in relative gene expression are highly significant (*p* < 0.0001 to *p* < 0.0072), as shown by gene set enrichment analysis (Supporting Information Fig. 2). We validated the expression of several T_RM_ signature genes by immunofluorescence and quantitative PCR. We had previously shown by qPCR that CD69^+^ memory T lymphocytes of murine bone marrow do not express S1pr1 5, 7, and show here that they also do not express Klf2 (Supporting Information Fig. 3A). In addition, human and murine CD69^+^ memory CD4^+^ T cells express CXCR6, but little CD62L (Supporting Information Fig. 3B and C). We also compared the expression of the human homologues from the murine T_RM_ signature genes by human bone marrow memory CD4^+^ T cells by principal‐component analysis. The predominant principal component 1 (PC1, 63%)) separates CD69^−^CD4^+^ cells of peripheral blood, when activated, from both, CD69^−^CD4^+^ cells of blood and bone marrow and CD69^+^CD4^+^ resting cells from bone marrow. PC2 (14%) then separates bone marrow CD69^+^CD4^+^ cells from CD69^−^CD4^+^ cells from both blood and bone marrow (Fig. 1D). Taken together, murine and human CD69^+^ memory T cells of the bone marrow express the T_RM_ signature genes reported for T_RM_ of other tissues 8, 9, suggesting that memory T cells are indeed resident in the bone marrow. Whether or not the CD69^−^ memory T cells of the bone marrow are also resident remains to be shown, e.g. by comparing their repertoire to that of circulating memory T cells from blood. The difference in gene expression between CD69^+^ and CD69^−^ memory T cells probably also explains why Baliu‐Pique and colleagues were not able to detect any clear T_RM_ signatures in total memory T cells isolated from goat bone marrow 1. Similarly, a lack of discrimination between resting memory T cells and their (proliferating) precursors may have impacted on their lifetime analysis by deuterium labeling. An alternative explanation would be that indeed the maintenance of T‐cell memory is differently organized in goats versus mice and humans.

**Figure 1:**
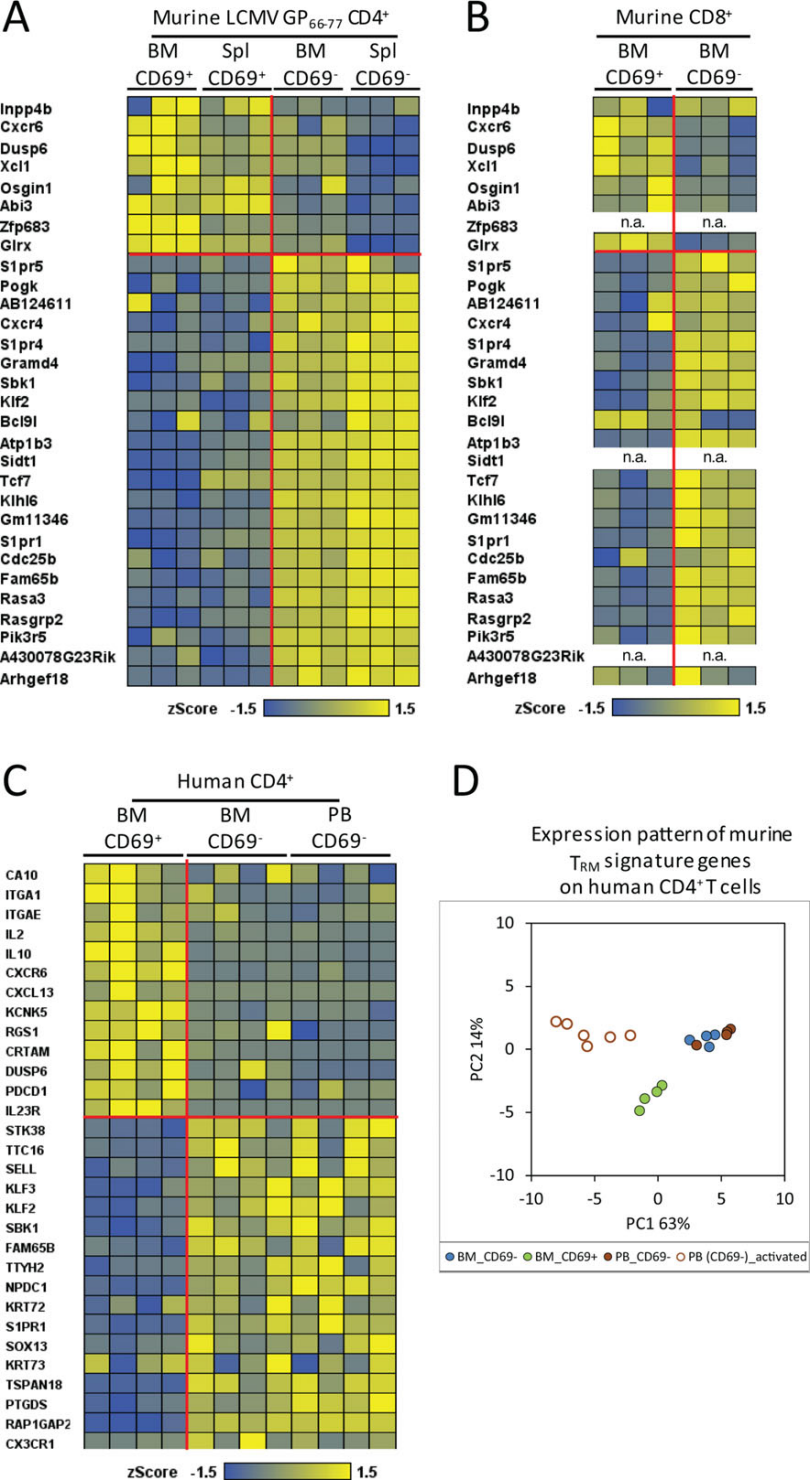
CD69 expression of bone marrow and spleen memory T cells defines a transcriptionally distinct population as reported earlier for T_RM_ cells of other tissues. (A and B) Heatmaps showing reported murine T_RM_ signature genes of other tissues for CD8 T cells 9, on bone marrow and spleen LCMV‐specific memory CD4^+^ T cells (A) and steady‐state bone marrow memory CD8^+^ T cells (B) isolated from C57BL/6 mice (Supporting Information Fig. 1; 5, 7). n.a., (probes) not present on the arrays, and thus gene expression not analyzed. (C) Heatmap of reported human T_RM_ signature genes for both CD4^+^ and CD8^+^ T cells of other tissues 8 on human bone marrow and paired peripheral blood (>98% being CD69^−^) memory CD4^+^ T cells according to CD69 expression 3. (D) Principal‐component analysis (PCA) of described murine T_RM_ signature genes 9 on the cells analyzed in C in comparison with recently activated blood effector/memory CD4^+^ T cells. In A–C, the horizontal and vertical red lines separate up‐ or downregulated genes as described for murine and human T_RM_ signature genes of other tissues 8, 9. (A) Data shown are from individual mice with a total of three mice from one experiment; (B) data shown are pooled from three independent experiments with cells pooled from 8 to 10 mice per experiment; C/D, data shown were taken from our previously published data GSE50677 3.

## Author contributions

Conceptualization, A.R. and J.D.; Methodology, F.S., M.A.M., Ö.S.A., and J.D.; Investigation, F.S., Ö.S.A., A.R., W.J.D., C.C., G.A.H. and M.F.M.; Formal Analysis, P.D.; Writing – Review & Editing, J.D. and A.R.; Visualization, P.D., F.S., W.J.D and J.D; Funding Acquisition, H.D.C., A.R. and J.D.

## Conflict of interest

The authors declare no commercial or financial conflict of interest.

## Data deposition

The data about resting murine memory CD4^+^ and CD8^+^ T lymphocytes discussed in this paper have been deposited in the Gene Expression Omnibus (GEO) database, www.ncbi.nlm.nih.gov/geo (accession no. GSE124796).

## Supporting information

Figure S1. Gating strategy for the isolation of LCMV‐specific memory CD4^+^ T cells, expressing or not CD69, from murine bone marrow (A) and spleen (B). Eight‐weekold C57BL/6 mice were twice immunized with LCMV GP_61‐80_, rested for 60 d, and LCMV.GP_66‐77_ tetramer‐specific CD69^+^ and CD69^‐^ memory CD4^+^ T cells were isolated by MACS enrichment of CD4^+^ cells and then by FACS. Data shown are representative of three different mice from one experiment.
Figure S2. Gene set enrichment analysis (GSEA) comparing gene sets of murine bone marrow/spleen CD69^+^ versus CD69‐ memory CD4^+^ T cells (A) and murine bone marrow CD69^+^ versus CD69^‐^ memory CD8^+^ T cells (B) using the published murine T_RM_ signature genes (Mackay et al., 2016), and gene sets of human bone marrow CD69^+^ versus bone marrow/blood CD69^‐^ memory CD4^+^ T cells using published human T_RM_ signature genes (Kumar et al., 2017). In each plot, the x axis shows the genes ranked with absolute value of log fold change between CD69^+^ versus CD69^‐^ cells, and the y axis shows the running enrichment score (ES), comparing the respective T_RM_ genes with indicated p values. Up: up‐regulated T_RM_ signature genes. Down: down‐regulated T_RM_ signature genes.
Figure S3. Validation of differential expression of the transcription factor Klf2 and the surface proteins by memory CD4^+^ T cells of bone marrow, spleen, and/or blood, expressing CD69 or not. A, Klf2 relative expression by LCMV‐specific CD69^+^ and CD69^‐^ memory CD4^+^ T cells of the bone marrow. Data shown are one experiment from individual mice with n = 3. B, CXCR6 surface protein expression by LCMVspecific CD69^+^ bone marrow and spleen and CD69‐ bone marrow memory CD4^+^ T cells. Geometric mean of fluorescent intensity (gMFI) of each analyzed cell subset is indicated. Data shown are representative of three independent experiments. C, CXCR6 and CD62L surface protein expression by human memory CD4^+^ and CD8^+^ T cells, expressing or not CD69, form paired bone marrow and peripheral blood samples. Data shown are one representative of three independent experiments.

## References

[1] Baliu‐Pique, M. et al., Front. Immunol. 2018 9: 2054. 30254637 10.3389/fimmu.2018.02054 PMC6141715

[2] Tokoyoda, K. et al., Immunity 2009 30: 721–730. 19427242 10.1016/j.immuni.2009.03.015

[3] Okhrimenko, A. et al., Proc. Natl. Acad. Sci. USA 2014 111: 9229–9234. 24927527 10.1073/pnas.1318731111 PMC4078840

[4] Chang, H. D. et al., Immunol. Rev. 2018 283: 86–98. 29664564 10.1111/imr.12656 PMC5947123

[5] Sercan Alp, Ö. et al., Eur. J. Immunol. 2015 45: 975–987. 25639669 10.1002/eji.201445295 PMC4415462

[6] Siracusa, F. et al., Eur. J. Immunol. 2017 47: 1900–1905. 28815584 10.1002/eji.201747063 PMC5698754

[7] Siracusa, F. et al., Proc. Natl. Acad. Sci. USA 2018 115: 1334–1339. 29358404 10.1073/pnas.1715618115 PMC5819416

[8] Kumar, B. V. et al., Cell Rep. 2017 20: 2921–2934. 28930685 10.1016/j.celrep.2017.08.078 PMC5646692

[9] Mackay, L. K. et al., Science 2016 352: 459–463. 27102484 10.1126/science.aad2035

[10] Cossarizza, A. et al., Eur. J. Immunol. 2017 47: 1584–1797. 29023707 10.1002/eji.201646632 PMC9165548

