# Supplementary material for: CD69+ memory T lymphocytes of the bone marrow and spleen express the signature transcripts of tissue‐resident memory T lymphocytes
Source: Eur J Immunol. 2019 Jan 30;49(6):966–8. doi: 10.1002/eji.201847982 (PMC6563480; doi:10.1002/eji.201847982)
Supplement: Supplementary file 1 — Figure S1. Gating strategy for the isolation of LCMV‐specific memory CD4+ T cells, expressing or not CD69, from murine bone marrow (A) and spleen (B). Eight‐weekold C57BL/6 mice were twice immunized with LCMV GP61‐80, rested for 60 d, and LCMV.GP66‐77 tetramer‐specific CD69+ and CD69‐ memory CD4+ T cells were isolated by MACS enrichment of CD4+ cells and then by FACS. Data shown are representative of three different mice from one experiment. Figure S2. Gene set enrichment analysis (GSEA) comparing gene sets of murine bone marrow/spleen CD69+ versus CD69‐ memory CD4+ T cells (A) and murine bone marrow CD69+ versus CD69‐ memory CD8+ T cells (B) using the published murine TRM signature genes (Mackay et al., 2016), and gene sets of human bone marrow CD69+ versus bone marrow/blood CD69‐ memory CD4+ T cells using published human TRM signature genes (Kumar et al., 2017). In each plot, the x axis shows the genes ranked with absolute value of log fold change between CD69+ versus CD69‐ cells, and the y axis shows the running enrichment score (ES), comparing the respective TRM genes with indicated p values. Up: up‐regulated TRM signature genes. Down: down‐regulated TRM signature genes. Figure S3. Validation of differential expression of the transcription factor Klf2 and the surface proteins by memory CD4+ T cells of bone marrow, spleen, and/or blood, expressing CD69 or not. A, Klf2 relative expression by LCMV‐specific CD69+ and CD69‐ memory CD4+ T cells of the bone marrow. Data shown are one experiment from individual mice with n = 3. B, CXCR6 surface protein expression by LCMVspecific CD69+ bone marrow and spleen and CD69‐ bone marrow memory CD4+ T cells. Geometric mean of fluorescent intensity (gMFI) of each analyzed cell subset is indicated. Data shown are representative of three independent experiments. C, CXCR6 and CD62L surface protein expression by human memory CD4+ and CD8+ T cells, expressing or not CD69, form paired bone marrow and peripheral blood samples. Data show [file EJI-49-966-s001.pdf]

European Journal of Immunology

Supporting Information
for
DOI 10.1002/eji.201847982

Francesco Siracusa, Pawel Durek, Mairi A. McGrath, Özen Sercan-Alp, Anna Rao, Weijie Du, Carla Cendón, Hyun-Dong Chang, Gitta Anne Heinz, Mir-Farzin Mashreghi, Andreas Radbruch and Jun Dong

CD69^{+} memory T lymphocytes of the bone marrow and spleen express the signature transcripts of tissue-resident memory T lymphocytes

© 2019 The Authors. *European Journal of Immunology* published by WILEY-VCH Verlag GmbH & Co. KGaA, Weinheim.

Supporting Information

Mice.

All mice were purchased from Charles River and maintained under specific pathogen-free conditions in the mouse facility of the German Rheumatism Research Center Berlin (DRFZ). All animal protocols used in this study were approved according to the German federal laws on animal protection by the State of Berlin. Eight-week-old C57BL/6 male mice were either untreated for $CD8^+$ cell analysis, or immunized i.p. with 100 µg LCMV $GP_{61–80}$ (Genecust) coupled to MSA (Merck Millipore) and NP in 200 µL PBS for $CD4^+$ cell analysis. Immunized mice were boosted i.p. with 100 µg unconjugated LCMV $GP_{61–80}$. Sixty days after the secondary immunization mice were sacrificed and analyzed.

Human Subjects.

The study protocol was reviewed and approved by the local ethical committee and informed consent obtained in accordance with the Declaration of Helsinki. We obtained bone marrow and matched peripheral blood samples from anonymous, systemically healthy adults (50-70-y; n = 4; 2 females, 2 males), undergoing joint replacement operation. Fresh samples were immediately prepared for analysis. Mononuclear cells of blood and bone marrow were isolated by density gradient sedimentation, using Ficoll-Hypaque (Sigma-Aldrich).

Flow Cytometry and Cell Sorting.

Flow cytometry and cell sorting were performed as described and consented in [1]. Single-cell suspensions were obtained from spleen, BM and/or blood samples.
Murine samples:
For staining, cells were pre-stained with anti-FcγRII/III (2.4G2) in FACS buffer (PBS/0.1%BSA/2 mM EDTA) for 10 min at 4 °C. For CD4 analysis, cells were then incubated in complete RPMI medium (Thermo Fisher) supplemented with 10% FCS for 60 min at 37 °C with 6 µg/mL LCMV.GP66–77 (DIYKGVYQFKSV) loaded tetramer or hCLiP control. Cells were further stained with surface markers for 15 min at 4 °C. For CD8 analysis, $CD8^+$ cells were enriched by magnetic cell sorting (MACS) using anti-CD8α microbeads (Miltenyi Biotech) prior to cell sorting. Viability of cells was assessed via fixable live/dead dye aqua (Thermo Fisher Scientific). The following antibodies were used: LCMV.$GP_{66–77}$ (DIYKGVYQFKSV) loaded tetramer or hCLiP control (NIH tetramer core facility), anti-CD3 (17A2 or 145-2C11), anti-CD4 (RM4.4), anti-CD44 (IM7), anti-B220 (RA3.6B2), anti-CD8 (53-6.72), anti-Gr1 (RB6-8C5), anti-CD69 (H1.2F3), anti-MHC class II (M5/114.15.2), anti-TCR Vα2 (B20.1), anti-CD8α (53-6.7), anti-CD45 (30-F11), anti-Ter119 (Ter119).
Human samples:
For staining, cells were incubated with FcR blocking reagent (Miltenyi Biotech) in FACS buffer (PBS/0.1%BSA/2 mM EDTA) for 10 min at 4 °C. Cells were then stained for 10 min at 4°C for surface staining. The following antibodies were used: anti-CD3 (SK7), anti-CD4 (TT1), anti-CD8 (RPA-TB), anti-CD45RO (UCHL1), anti-CD69 (FN50), anti-CD19 (BV12), anti-CD14 (TM1), anti-CD62L/SELL (DREG-56), and anti-CXCR6 (K041E5). Viability of cells was assessed via DAPI (Thermo Fisher Scientific).

For cell sorting, a FACS Aria I (BD Biosciences) cell sorter was used. Flow cytometric data were analyzed using FlowJo software (FlowJo LLC).

Whole-Transcriptome Profiling by RNA-Seq.

Total RNA from purified tetramer$^+$ memory CD4$^+$ T cells isolated from spleen and BM was extracted using a miRNeasy Micro Kit (Qiagen), according to the manufacturer's instructions. RNA quality was determined on a Bioanalyzer using the RNA 6000 Pico Kit (Agilent) prior to mRNA-specific library preparations. cDNA synthesis and library completion were performed as described earlier [2]. Paired-end (2 × 75 nucleotides) sequencing was performed on a NextSeq500 using a midoutput flow cell. Sequence reads were mapped to mouse GRCm38/mm10 genome with TopHat2 [3] in very-sensitive settings for Bowtie2 [4] and Ensemble annotation release 67. Gene expression was quantified by HTSeq [5] and analyzed using DESeq2 [6]. Signatures for T_{RM}s, as defined by MacKay et al [7] for CD8 T lymphocytes and innate cells, were visualized by heatmaps. The order of genes and samples were set manually to facilitate the comparison to previous publications. Sequencing data are deposited in NCBI's Gene Expression Omnibus (GEO), www.ncbi.nlm.nih.gov/geo (accession no. GSE124796).

Whole-Transcriptome Profiling by Microarray Analysis.

Total RNA of murine CD8$^+$ T cells was extracted using a NucleoSpin RNA XS Kit (Macherey-Nagel) according to the manufacturer's recommendations. Gene expression was analyzed using MG_U430_2 GeneChips (Affymetrix), according to the manufacturer's recommendations. The integrity and amount of isolated RNA was assessed for each sample using an Agilent 2100 Bioanalyzer (Agilent, Waldbronn, Germany) and a NanoDrop ND-1000 spectrophotometer (NanoDrop Technologies, Wilmington, DE). The microarray analysis of gene expression was performed in house at the DRFZ as described before [8]. Gene expression was quantified by RMA using the affy R algorithm [9]. Signatures for resident cells, as defined by MacKay et al for murine and Kumar et al for human, were visualized by heatmaps. The order of genes and samples were set manually to facilitate the comparison to previous publications [7, 10]. The Principle Component Analysis (PCA) was based on signature genes identified by Kumar et al [10], and based on log transformed expression values. The murine CD8 data are deposited in NCBI's GEO (accession no. GSE124796). The human data discussed in this paper were taken from GEO series accession number GSE50677 [11].

Gene Set Enrichment Analysis.

Gene Set Enrichment Analysis (GSEA) [12] was performed using the GSEA software, based on signatures for gene expression of which is up- and down-regulated in resident cells, as defined by MacKay et al for murine CD8 T lymphocytes and innate cells [7] and by Kumar et al for human memory CD4 and CD8 T lymphocytes from lung and spleen [10].

qPCR Analysis.

cDNA obtained from FACS sorted CD69$^+$ and CD69$^-$ LCMV-specific memory CD4$^+$ T cells isolated from BM was quantified by TaqMan Gene Expression Assay-based real-time PCR, using probes for hypoxanthine guanine phosphoribosyl transferase (hprt) (assay # Mm03024075_m1) and Krüppel-like factor 2 (Klf-2) (assay #

Mm00500486_g1). Expression values of Klf2 were normalized to values of Hprt by the change-in-threshold method (2-DCt).

References:

1 **Cossarizza, A., Chang, H. D., Radbruch, A., Akdis, M., Andra, I., Annunziato, F., Bacher, P., Barnaba, V., Battistini, L., Bauer, W. M., Baumgart, S., Becher, B., Beisker, W., Berek, C., Blanco, A., Borsellino, G., Boulais, P. E., Brinkman, R. R., Buscher, M., Busch, D. H., Bushnell, T. P., Cao, X., Cavani, A., Chattopadhyay, P. K., Cheng, Q., Chow, S., Clerici, M., Cooke, A., Cosma, A., Cosmi, L., Cumano, A., Dang, V. D., Davies, D., De Biasi, S., Del Zotto, G., Della Bella, S., Dellabona, P., Deniz, G., Dessing, M., Diefenbach, A., Di Santo, J., Dieli, F., Dolf, A., Donnenberg, V. S., Dorner, T., Ehrhardt, G. R. A., Endl, E., Engel, P., Engelhardt, B., Esser, C., Everts, B., Dreher, A., Falk, C. S., Fehniger, T. A., Filby, A., Fillatreau, S., Follo, M., Forster, I., Foster, J., Foulds, G. A., Frenette, P. S., Galbraith, D., Garbi, N., Garcia-Godoy, M. D., Geginat, J., Ghoreschi, K., Gibellini, L., Goettlinger, C., Goodyear, C. S., Gori, A., Grogan, J., Gross, M., Grutzkau, A., Grummitt, D., Hahn, J., Hammer, Q., Hauser, A. E., Haviland, D. L., Hedley, D., Herrera, G., Herrmann, M., Hiepe, F., Holland, T., Hombrink, P., Houston, J. P., Hoyer, B. F., Huang, B., Hunter, C. A., Iannone, A., Jack, H. M., Javega, B., Jonjic, S., Juelke, K., Jung, S., Kaiser, T., Kalina, T., Keller, B., Khan, S., Kienhofer, D., Kroneis, T.,** Guidelines for the use of flow cytometry and cell sorting in immunological studies. *Eur J Immunol* 2017. **47**: 1584-1797.

2 **Siracusa, F., Alp, O. S., Maschmeyer, P., McGrath, M., Mashreghi, M. F., Hojyo, S., Chang, H. D., Tokoyoda, K. and Radbruch, A.,** Maintenance of CD8(+) memory T lymphocytes in the spleen but not in the bone marrow is dependent on proliferation. *Eur J Immunol* 2017. **47**: 1900-1905.

3 **Kim, D., Pertea, G., Trapnell, C., Pimentel, H., Kelley, R. and Salzberg, S. L.,** TopHat2: accurate alignment of transcriptomes in the presence of insertions, deletions and gene fusions. *Genome Biol* 2013. **14**: R36.

4 **Langmead, B. and Salzberg, S. L.,** Fast gapped-read alignment with Bowtie 2. *Nat Methods* 2012. **9**: 357-359.

5 **Liao, Y., Smyth, G. K. and Shi, W.,** featureCounts: an efficient general purpose program for assigning sequence reads to genomic features. *Bioinformatics* 2014. **30**: 923-930.

6 **Love, M. I., Huber, W. and Anders, S.,** Moderated estimation of fold change and dispersion for RNA-seq data with DESeq2. *Genome Biol* 2014. **15**: 550.

7 **Mackay, L. K., Minnich, M., Kragten, N. A., Liao, Y., Nota, B., Seillet, C., Zaid, A., Man, K., Preston, S., Freestone, D., Braun, A., Wynne-Jones, E., Behr, F. M., Stark, R., Pellicci, D. G., Godfrey, D. I., Belz, G. T., Pellegrini, M., Gebhardt, T., Busslinger, M., Shi, W., Carbone, F. R., van Lier, R. A., Kallies, A. and van Gisbergen, K. P.,** Hobit and Blimp1 instruct a universal transcriptional program of tissue residency in lymphocytes. *Science* 2016. **352**: 459-463.

8 **Tokoyoda, K., Zehentmeier, S., Hegazy, A. N., Albrecht, I., Grun, J. R., Lohning, M. and Radbruch, A.,** Professional memory CD4+ T lymphocytes preferentially reside and rest in the bone marrow. *Immunity* 2009. **30**: 721-730.

9 **Gautier, L., Cope, L., Bolstad, B. M. and Irizarry, R. A.,** affy--analysis of Affymetrix GeneChip data at the probe level. *Bioinformatics* 2004. **20**: 307-315.

10 **Kumar, B. V., Ma, W., Miron, M., Granot, T., Guyer, R. S., Carpenter, D. J., Senda, T., Sun, X., Ho, S. H., Lerner, H., Friedman, A. L., Shen, Y. and Farber, D. L.,** Human Tissue-Resident Memory T Cells Are Defined by Core Transcriptional and Functional Signatures in Lymphoid and Mucosal Sites. *Cell Rep* 2017. **20**: 2921-2934.

11 **Okhrimenko, A., Grun, J. R., Westendorf, K., Fang, Z., Reinke, S., von Roth, P., Wassilew, G., Kuhl, A. A., Kudernatsch, R., Demski, S., Scheibenbogen, C., Tokoyoda, K., McGrath, M. A., Raftery, M. J., Schonrich, G., Serra, A., Chang, H.**

D., Radbruch, A. and Dong, J., Human memory T cells from the bone marrow are resting and maintain long-lasting systemic memory. *Proc Natl Acad Sci U S A* 2014. **111**: 9229-9234.

12 **Subramanian, A., Tamayo, P., Mootha, V. K., Mukherjee, S., Ebert, B. L., Gillette, M. A., Paulovich, A., Pomeroy, S. L., Golub, T. R., Lander, E. S. and Mesirov, J. P.,** Gene set enrichment analysis: a knowledge-based approach for interpreting genome-wide expression profiles. *Proc Natl Acad Sci U S A* 2005. **102**: 15545-15550.

Supporting Information Figure S1

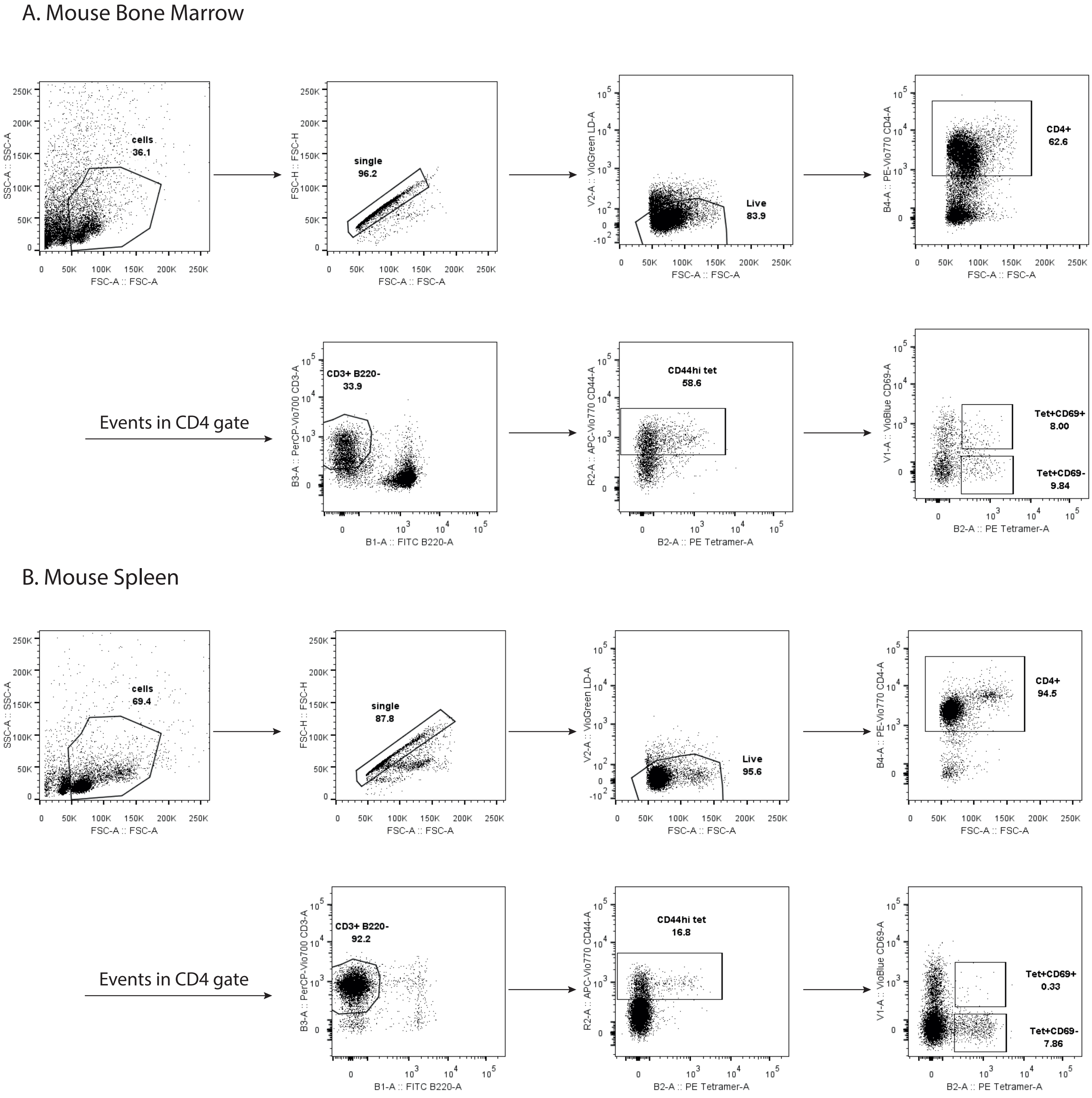

Figure S1. Gating strategy for the isolation of LCMV-specific memory $CD4^+$ T cells, expressing or not CD69, from murine bone marrow (A) and spleen (B). Eight-week-old C57BL/6 mice were twice immunized with LCMV $GP_{61-80,}$ rested for 60 d, and LCMV.GP_{66-77} tetramer-specific $CD69^+$ and $CD69^-$ memory $CD4^+$ T cells were isolated by MACS enrichment of $CD4^+$ cells and then by FACS. Data shown are representative of three different mice from one experiment.

Supporting Information Figure S2

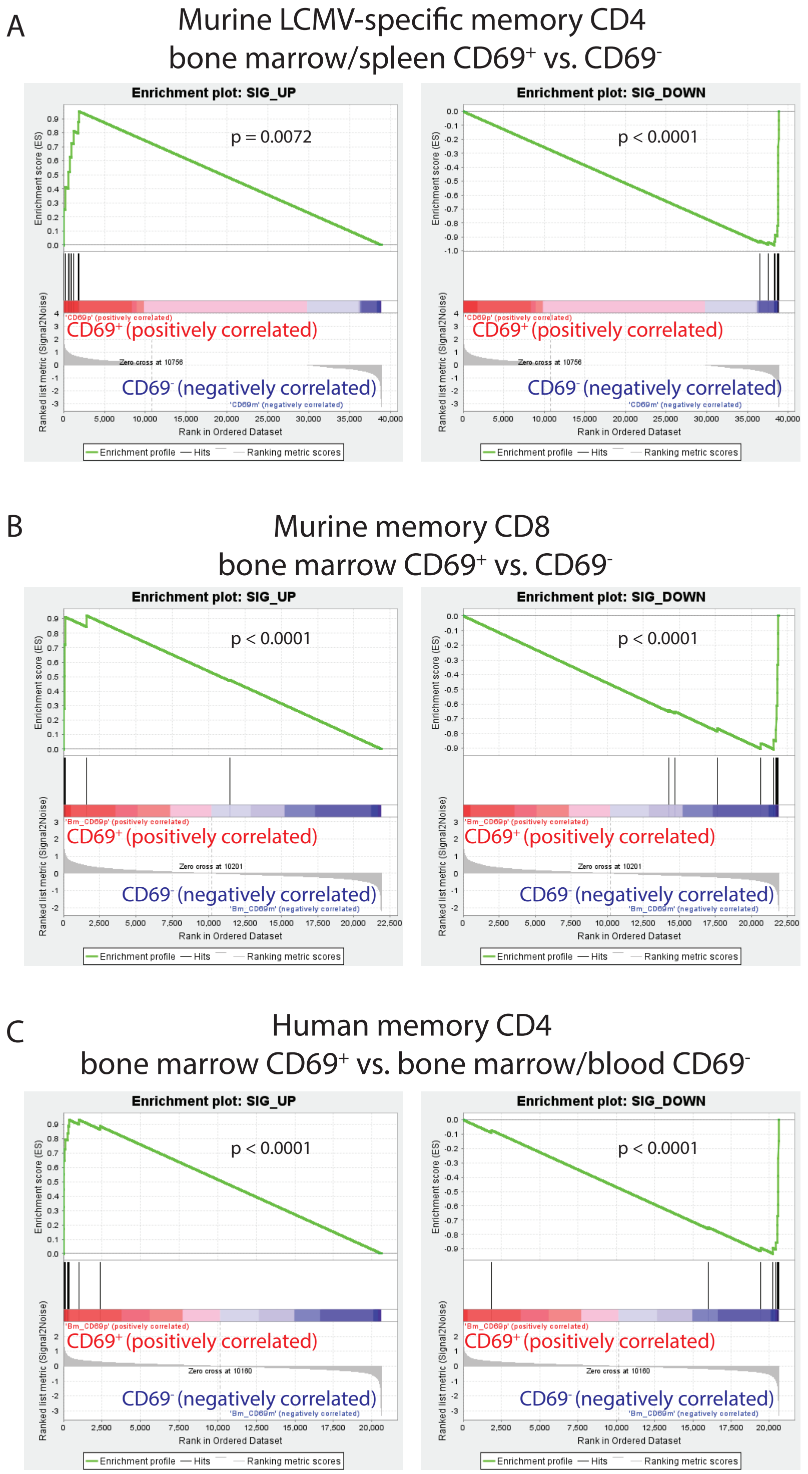

Figure S2. Gene set enrichment analysis (GSEA) comparing gene sets of murine bone marrow/spleen CD69$^+$ versus CD69$^-$ memory CD4$^+$ T cells (A) and murine bone marrow CD69$^+$ versus CD69$^-$ memory CD8$^+$ T cells (B) using the published murine T_{RM} signature genes (Mackay et al., 2016), and gene sets of human bone marrow CD69$^+$ versus bone marrow/blood CD69$^-$ memory CD4$^+$ T cells using published human T_{RM} signature genes (Kumar et al., 2017). In each plot, the x axis shows the genes ranked with absolute value of log fold change between CD69$^+$ versus CD69$^-$ cells, and the y axis shows the running enrichment score (ES), comparing the respective T_{RM} genes with indicated p values. Up: up-regulated T_{RM} signature genes. Down: down-regulated T_{RM} signature genes.

Supporting Information Figure S3

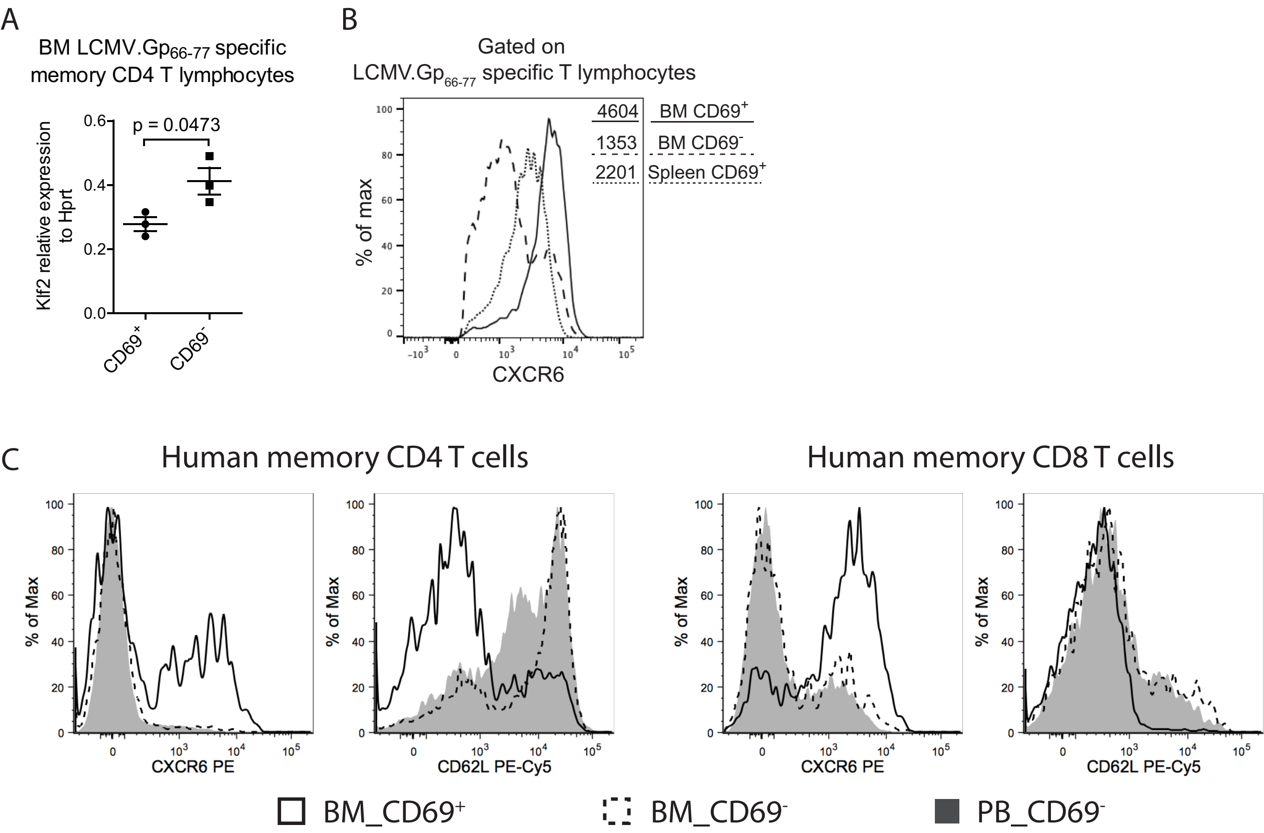

Figure S3. Validation of differential expression of the transcription factor Klf2 and the surface proteins by memory $CD4^+$ T cells of bone marrow, spleen, and/or blood, expressing CD69 or not. A, Klf2 relative expression by LCMV-specific $CD69^+$ and $CD69^-$ memory $CD4^+$ T cells of the bone marrow. Data shown are one experiment from individual mice with n = 3. B, CXCR6 surface protein expression by LCMV-specific $CD69^+$ bone marrow and spleen and $CD69^-$ bone marrow memory $CD4^+$ T cells. Geometric mean of fluorescent intensity (gMFI) of each analyzed cell subset is indicated. Data shown are representative of three independent experiments. C, CXCR6 and CD62L surface protein expression by human memory $CD4^+$ and $CD8^+$ T cells, expressing or not CD69, form paired bone marrow and peripheral blood samples. Data shown are one representative of three independent experiments.
